# *In vacuo* X-ray data collection from graphene-wrapped protein crystals

**DOI:** 10.1107/S1399004715014194

**Published:** 2015-09-26

**Authors:** Anna J. Warren, Adam D. Crawshaw, Jose Trincao, Pierre Aller, Simon Alcock, Ioana Nistea, Paula S. Salgado, Gwyndaf Evans

**Affiliations:** a Diamond Light Source, Harwell Science and Innovation Campus, Didcot OX11 0DE, England; b Institute for Cell and Molecular Biosciences, Faculty of Medical Sciences, Newcastle University, Newcastle upon Tyne NE2 4HH, England

**Keywords:** vacuum, graphene, room temperature, dehydration

## Abstract

The measurement of diffraction data from macromolecular crystal samples held *in vacuo* holds the promise of a very low X-ray background and zero absorption of incident and scattered beams, leading to better data and the potential for accessing very long X-ray wavelengths (>3 Å) for native sulfur phasing. Maintaining the hydration of protein crystals under vacuum is achieved by the use of liquid jets, as with serial data collection at free-electron lasers, or is side-stepped by cryocooling the samples, as implemented at new synchrotron beamlines. Graphene has been shown to protect crystals from dehydration by creating an extremely thin layer that is impermeable to any exchanges with the environment. Furthermore, owing to its hydrophobicity, most of the aqueous solution surrounding the crystal is excluded during sample preparation, thus eliminating most of the background caused by liquid. Here, it is shown that high-quality data can be recorded at room temperature from graphene-wrapped protein crystals in a rough vacuum. Furthermore, it was observed that graphene protects crystals exposed to different relative humidities and a chemically harsh environment.

## Abstract 2

A method is reported for collecting room-temperature data from protein crystals under vacuum by protecting them with a thin graphene layer.

## 1. Introduction   

Third-generation synchrotron sources have created new opportunities for the measurement of macromolecular crystallography (MX) diffraction data on ever more challenging structural targets, yielding much smaller and weakly diffracting crystals. This is owing to developments in beamline instrumentation, such as pixel-array detector (PAD) technology (Mueller *et al.*, 2012 ▸), improved quality of focusing optics (Duke & Johnson, 2010 ▸) and higher brightness sources with greater stability. The introduction of microfocus MX beamlines has also helped immensely in this area, satisfying the ever-increasing demand to probe the smallest crystals possible (Evans, Axford, Waterman *et al.*, 2011 ▸; Smith *et al.*, 2012 ▸; Duran *et al.*, 2013 ▸; Fischetti *et al.*, 2013 ▸; Hirata *et al.*, 2013 ▸; Schneider *et al.*, 2013 ▸; Holton & Frankel, 2010 ▸).

As crystal volumes decrease, the diffraction intensity decreases proportionally and sources of X-ray background other than the crystal can bury the signal. This limits the maximum resolution obtainable from small crystals. Matching the beam size to the crystal size has been shown to dramatically increase the signal to noise observed by reducing scatter from surrounding material, increasing the resolution at which reflections are detected (Evans, Axford & Owen, 2011 ▸), but other sources of background scatter may still exist. Major sources of X-ray background include the solvent surrounding the crystal and the crystal mount or loop. Crystal mounts have been adapted to include a drainage channel to help remove any excess liquor around the crystal, whilst being constructed from polyimide to contribute minimally to background scatter (Thorne *et al.*, 2003 ▸). The air paths before and after the sample, up until the X-ray beamstop, or the cryo-gas stream, also contribute significantly to the background scatter. A reduction in air scatter has been demonstrated for microcrystals on improvements to the collimation and by replacing nitrogen with helium in the cold gas stream (Glaeser *et al.*, 2000 ▸). Further enhancements have also been gained through close alignment of the apertures and beamstop to within just a few millimetres of the sample position (Madsen *et al.*, 1999 ▸). Instrument-generated scatter from slits, apertures or optics may also add to the background noise observed at the detector.

It has been shown that measurement of diffraction data where the crystals are surrounded by a helium or evacuated atmosphere greatly reduces the background observed on the detector (Perutz & Rogers, 1946 ▸; Krieger & Stroud, 1976 ▸; Liu *et al.*, 2001 ▸; Hirata *et al.*, 2013 ▸; Djinović Carugo *et al.*, 2005 ▸; Hendrickson & Ogata, 1997 ▸). However, the study of single crystals under vacuum has been limited to those samples that can survive at room temperature within the vacuum chamber (Lin *et al.*, 2011 ▸). An evacuated sample environment is currently used at the CXI beamline (Boutet & Williams, 2010 ▸) at the Linac Coherent Light Source (LCLS) to perform serial femtosecond crystallography (SFX; Chapman *et al.*, 2011 ▸) measurements from crystals delivered to the beam in a hydrated state using a gas dynamic virtual nozzle (GDVN; Weierstall *et al.*, 2012 ▸). Synchrotron MX beamlines are also now being developed in which both the incident and diffracted beam paths from source to detector have been placed under vacuum (Mykhaylyk & Wagner, 2013 ▸). This allows the use of longer wavelengths to access the absorption edges of atoms such as sulfur and phosphorus for anomalous diffraction phasing experiments. At these wavelengths, the vacuum is vital in reducing air absorption and scatter (Lehmann *et al.*, 1993 ▸; Mykhaylyk & Wagner, 2013 ▸).

A major obstacle to placing protein crystals under vacuum is incompatibility with the use of open-flow cryocooling or humidity-control devices (Mykhaylyk & Wagner, 2013 ▸). This means that protein crystals are prone to dehydration and, ultimately, deterioration of diffraction integrity. Direct cooling of the sample stage in vacuum is used in cryo-electron microscopy and cryo-electron crystallography, where rotation of the sample stage is typically limited to ±70° (Nannenga *et al.*, 2014 ▸). This technology has not yet been successfully applied to X-ray crystallography, where complete rotations of the sample stage are typically required.

The recent renaissance in room-temperature MX (Axford *et al.*, 2012 ▸; Bingel-Erlenmeyer *et al.*, 2011 ▸) partnered with increased crystal lifetime using high dose-rate/frame-rate data collection (Owen *et al.*, 2014 ▸) could benefit from methodology preventing crystal dehydration *in vacuo*. One method for preventing dehydration is to use an adhesive or an oil to coat crystals (Mazzorana *et al.*, 2014 ▸); however, this can act to increase the background on the diffraction images and counter the motivation for using a vacuum in the first instance.

The use of graphene as a crystal-mounting platform for protein crystals has recently been reported (Wierman *et al.*, 2013 ▸). Graphene has been shown to have many distinct properties, including a complete impermeability to gases (Bunch *et al.*, 2008 ▸). This makes it an attractive material for protecting protein crystals from dehydration. Wierman and coworkers reported the usefulness of graphene in reducing the observed X-ray background whilst maintaining the hydration of protein crystals once wrapped. The mounting procedure reduced mother liquor around the crystal as well as being only a few nanometres thick to help reduce background scatter. It was also possible to prevent crystal dehydration for up to 10 min at room temperature in air before cryocooling for data collection (Wierman *et al.*, 2013 ▸).

Here, we further investigate the use of graphene as a protective wrap for protein crystals exposed to a number of different environments. We have used graphene to wrap crystals before placing them in a rough vacuum for room-temperature data collection and tested its protective properties against variable humidity and chemically harsh environments. Our experiments are carried out on relatively large crystals (50–200 µm) to establish a proof of principle for using graphene in this way. Our results demonstrate that graphene acts to maintain crystal hydration during exposure to these harsh conditions, allowing the measurement of high-quality diffraction data at room temperature.

## 2. Materials and methods   

### 2.1. Sample crystals   

#### 2.1.1. Crystallization of lysozyme   

Commercial lyophilized lysozyme from hen egg white (Sigma–Aldrich) was resuspended in Milli-Q water to a concentration of 100 mg ml^−1^. Sitting drops were made by mixing 1 µl lysozyme solution and 1 µl reservoir solution [20%(*w*/*v*) polyethylene glycol (PEG) monomethyl ether (MME) 5000, 1 *M* NaCl, 50 m*M* sodium acetate pH 4.5] and were equilibrated against 300 µl reservoir solution at 20°C. Crystals (100 × 100 × 50 µm) grew within 3 h and were found to belong to space group *P*4_3_2_1_2, with unit-cell parameters *a* = *b* ≃ 78, *c* ≃ 38 Å. The crystals did not require cryoprotection before being flash-cooled in liquid nitrogen.

#### 2.1.2. Crystallization of glucose isomerase   

Commercial glucose isomerase (GI) from *Streptomyces murinus* (Sigma–Aldrich) was resuspended in solution [10 m*M* 4-(2-hydroxy­ethyl)-1-piperazineethanesulfonic acid (HEPES) pH 7.0, 1 m*M* MgCl_2_] to a concentration of 16.3 mg ml^−1^. Sitting drops were made by mixing 2 µl protein solution and 2 µl reservoir solution [100 m*M* HEPES pH 7.0, 100 m*M* MgCl_2_, 10%(*v*/*v*) PEG 400, 28%(*w*/*v*) glucose] and were equilibrated against 300 µl reservoir solution at 20°C. Crystals (120 × 80 × 80 µm) grew within 7 d and were found to belong to space group *I*222, with unit-cell parameters *a* ≃ 93, *b* ≃ 98, *c* ≃ 103 Å. The cystals did not require cryoprotection before being flash-cooled in liquid nitrogen.

#### 2.1.3. Crystallization of thaumatin   

Commercial thaumatin from *Thaumatococcus danielli* (Sigma–Aldrich) was resuspended in Milli-Q water to a concentration of 40 mg ml^−1^. Sitting drops were made by mixing 4 µl protein solution and 2 µl reservoir solution [50 m*M* *N*-(2-acetamido)iminodiacetic acid (ADA) pH 6.8, 600 m*M* potassium sodium tartrate, 20%(*v*/*v*) glycerol] and were equilibrated against 500 µl reservoir solution at 20°C. Crystals (200 × 200 × 100 µm) grew within 7 d and were found to belong to space group *P*4_1_2_1_2, with unit-cell parameters *a* = *b* ≃ 59, *c* ≃ 151 Å. The crystals did not require cryoprotection before being flash-cooled in liquid nitrogen.

### 2.2. Mounting of crystals in graphene   

**Figure 1:**
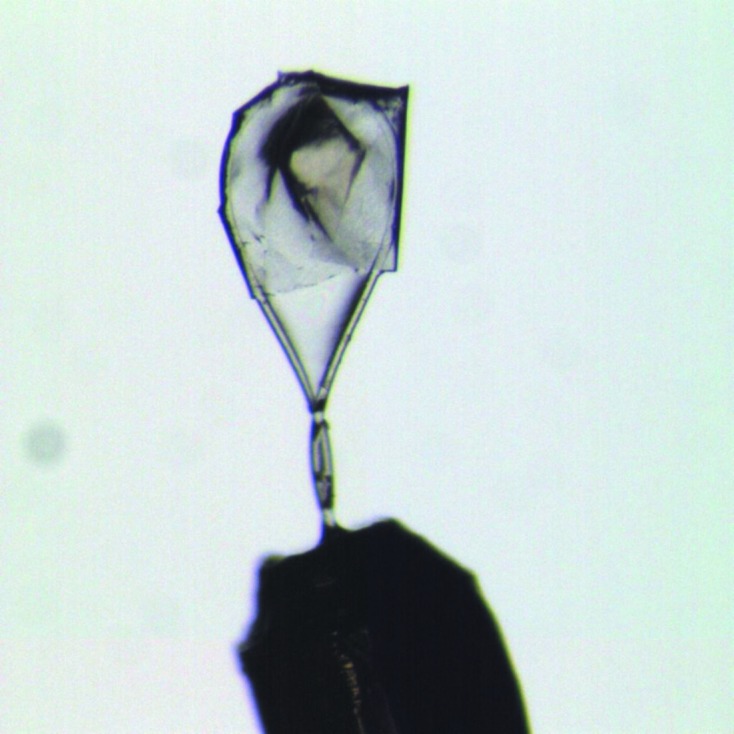
An optical image of a crystal of thaumatin wrapped in graphene/PMMA mounted within a nylon loop.

The graphene used in this investigation was Trivial Transfer graphene purchased from ACS Material. It is supplied as 3–5 layers of graphene spin-coated with poly(methylmethacrylate) (PMMA), with a quoted PMMA thickness of ∼500 nm (http://acsmaterial.com). The thickness of the PMMA was measured by micro-interferometry on a Bruker Contour GT-X and was found to be 100 ± 10 nm (Supplementary Fig. S1). It is possible to entirely remove the PMMA by pipetting a few drops of acetone onto the completely dry graphene/PMMA, ensuring that the surface of the graphene/PMMA is completely covered. The removal of the PMMA layer was confirmed by infrared spectroscopy and micro-interferometry (Supplementary Figs. S2 and S3). However, without support the remaining graphene layers are too fragile and are destroyed if further manipulated (Suk *et al.*, 2011 ▸). Owing to the delicate nature of this graphene, it was used as supplied for the mounting of crystals. Crystals were transferred from the crystallization drop to a custom loop containing mother liquor by pipetting a few of the crystals into the loop (Supplementary Fig. S4). Crystals were mounted in graphene/PMMA following the previously reported protocol (Wierman *et al.*, 2013 ▸). Fig. 1 ▸ shows an optical image of a thaumatin crystal wrapped in graphene/PMMA. Further details of the procedure can be found in the Supporting Information. Wrapped crystals were subsequently flash-cooled, if required, or placed directly into the vacuum chamber, left in air or placed under vacuum at 2000 Pa. Further details of each experiment are given in §§2.3, 2.4 and 2.5.

### 2.3. Protection of graphene/PMMA-wrapped crystals from vacuum   

**Figure 2:**
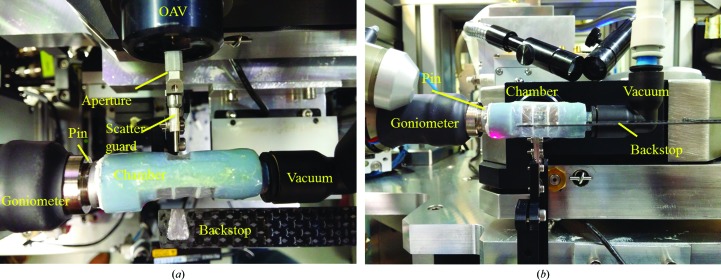
The experimental setup of the three-dimensionally printed vacuum chamber on beamline I04 at Diamond Light Source. (*a*) Viewed from above and (*b*) viewed from the side. The components of the setup have been labelled, where OAV indicates the on-axis viewing system.

In order to investigate whether graphene/PMMA can protect crystals at room temperature in vacuum, a simple chamber was designed in-house and then printed in three dimensions (3D Alchemy; Fig. 2 ▸). The entrance and exit windows of the chamber were covered in 5 µm thick Mylar sheets, which were glued in place using epoxy adhesive. The entrance window was approximately 7.5 × 6.5 mm and the exit window was approximately 15 × 15 mm, with added struts to support the Mylar window. These windows are large enough to allow a rotation range of 60° for data collection without visible shadowing of the diffracted beams. The vacuum path within the chamber was approximately 10.5 mm, with an overall air path between the windows and scatterguard and beamstop of approximately 3.5 mm. One end of the chamber was designed to take a SPINE standard pin, which was held in place with white tack. This then allowed the whole chamber to be mounted directly onto the magnetic goniometer on beamline I04 at Diamond Light Source. The other end had an 8 mm fitting connected to a vacuum pump *via* a vacuum gauge to measure the pressure within the chamber (diaphragm vacuum pump from KNF). In general, pumping took a few seconds to reach around 2000 Pa.

Thaumatin crystals were wrapped in graphene/PMMA and immediately placed into the vacuum chamber. This process took approximately 5 min. Data collections were carried out on the samples either under vacuum at 2000 Pa or in air at atmospheric pressure (but still within the chamber). As a control, one crystal of thaumatin was also placed under vacuum without a graphene/PMMA wrap.

### 2.4. Protection of graphene/PMMA-wrapped crystals from dehydration   

A high-precision crystal humidifier/dehumidifier (HC1b) from Arinax was used to expose the crystals to different relative humidities (Sanchez-Weatherby *et al.*, 2009 ▸).

Mounted crystals of GI and of GI wrapped in graphene/PMMA were prepared and placed in a humid airstream with 65% relative humidity. It has been shown that a phase transition (a change in space group from *I*222 to *P*2_1_2_1_2) occurs in dehydrated crystals of GI when exposed to a relative humidity of between 70 and 90% (Lobley *et al.*, 2015 ▸). A relative humidity of 65% was chosen to ensure that a complete transition had occurred. In this way, the absence of a phase transition in graphene/PMMA-wrapped crystals would be an indicator of the ability of graphene/PMMA to protect the crystals from dehydration. Crystals were left at this humidity for 5 min before flash-cooling in liquid nitrogen. As a comparison, several crystals of GI and of GI wrapped in graphene/PMMA were mounted and immediately flash-cooled. Data collections for these samples were carried out at 100 K in the open flow of the nitrogen-gas cryostream.

Mounted crystals of lysozyme were treated in the same way as described above for GI but using a relative humidity of 70%. It has been observed for lysozyme that below a relative humidity of 88% there is a deterioration in the crystal quality (Dobrianov *et al.*, 2001 ▸). A relative humidity of 70% was selected to guarantee that a decline in the quality of the data would be observed without protection from the graphene/PMMA.

### 2.5. Protection of graphene/PMMA-wrapped crystals from a chemically harsh environment   

The PMMA layer can be removed from graphene/PMMA; however, a backing material is required to support the multilayer graphene that remains. In this case, a MiTeGen MicroLoop was used as a support for the graphene. A crystal of lysozyme was mounted with graphene/PMMA following the procedure in §2.2. Once mounted, the graphene/PMMA-wrapped crystal was left for several minutes to ensure that the external surface was completely dry. The loop and wrapped crystal were soaked in acetone whilst monitoring them under a microscope to ensure, as far as possible, that the crystal remained wrapped. After leaving to soak for approximately 10 s, the sample was flash-cooled in liquid nitrogen ready for data collection. Data were collected in the open flow of the nitrogen-gas cryostream at 100 K. Soaking an unwrapped crystal of lysozyme and assessing the impact on diffraction quality established the 10 s soaking time. In this case, very poor diffraction was observed to typically ∼5 Å from an unwrapped crystal. The diffraction pattern was indicative of a highly mosaic crystal and proved difficult to index.

### 2.6. Data collection and processing   

Diffraction data were collected on beamline I04 at Diamond Light Source using an X-ray energy of 12.658 keV and a beam size of 90 × 45 µm (0.7 × 10^12^ photons s^−1^) or 20 × 20 µm (0.7 × 10^11^ photons s^−1^) (defined using apertures). Diffraction images were measured with a PILATUS 6M-F area detector. For samples within the vacuum chamber, data collections were limited to a total oscillation of approximately 60°, whereas crystals outside the chamber were exposed for a total oscillation of 180°. The oscillation range for all samples was 0.10° per image, with an exposure time of 40 ms per image. Crystals within the chamber were collected at approximately 295 K with a vacuum pressure of ∼2000 Pa. Samples outside the chamber were collected at 100 K cooled with an Oxford Cryosystems Cryostream.

X-ray diffraction data were analysed using *xia*2 (Winter *et al.*, 2013 ▸) with the -3dii option, which invokes the use of *XDS* (Kabsch, 2010 ▸) and *AIMLESS* (Evans & Murshudov, 2013 ▸).

Several data sets were measured for wrapped thaumatin crystals under vacuum. For brevity, the results for only one representative data set are displayed in Table 1 ▸, with the remainder provided in Supplementary Table S1. The crystals within the vacuum chamber were difficult to centre owing to the limited rotation range; each data set was therefore manually inspected to remove images with *R*_merge_ above 0.5. A resolution cutoff of *I*/σ(*I*) > 2 was applied to the data.

For the structure determination and refinement of wrapped thaumatin crystals under vacuum, a previously solved structure of thaumatin (Sauter *et al.*, 2002 ▸; PDB entry 1kwn) was stripped of all nonprotein atoms and was used for rigid-body refinement with *REFMAC*5 (Murshudov *et al.*, 2011 ▸). The structure refinement consisted of a cycle of model building with *Coot* (Emsley *et al.*, 2010 ▸) followed by restrained refinement with *REFMAC*5. The *R*_free_ value was calculated using 5% of the reflections put aside during the refinement. The last step of refinement included TLS modelling. The statistics after refinement are shown in Table 2 ▸. The deposition code for the final model in the Protein Data Bank is 4zxr.

For the HC1b experiments, at least three data sets were measured for each condition and the results in Table 3 ▸ and 4 ▸ use a representative data set for each scenario. The processing statistics for all of the data sets can be found in Supplementary Tables S2–S9. All lysozyme and GI data were processed to the edge of the detector (1.7 and 1.8 Å, respectively), except in the case where the crystals were dehydrated, where a resolution cutoff of *I*/σ(*I*) > 2 was applied.

For the crystals wrapped in graphene/PMMA and then exposed to acetone, the data were processed as above using a resolution cutoff of *I*/σ(*I*) > 2.

## 3. Results   

**Figure 3:**
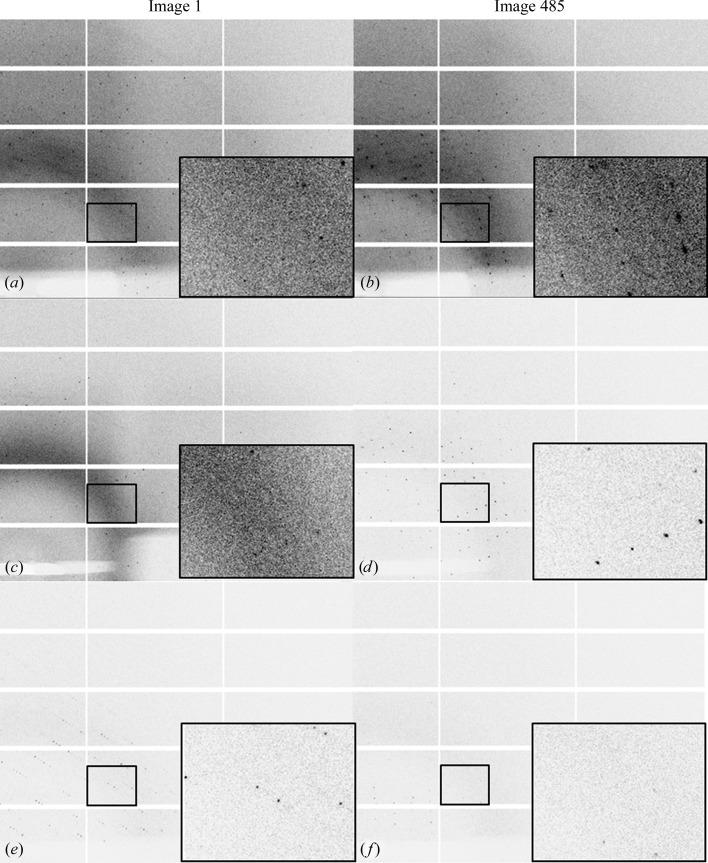
Diffraction patterns of thaumatin within the vacuum chamber; the inset in each pattern shows an enlarged area of the diffraction at an approximate resolution range of 6.4–3.7 Å. (*a*, *b*) Images 1 and 485, respectively, of crystals of thaumatin wrapped in graphene/PMMA within the chamber in air at atmospheric pressure. (*c*, *d*) Images 1 and 485, respectively, of crystals of thaumatin wrapped in graphene/PMMA within the vacuum chamber under vacuum. (*e*, *f*) Images 1 and 485, respectively, of crystals of thaumatin within the vacuum chamber under vacuum. All images are displayed with the same contrast levels; (*a*) and (*b*) use a 90 × 45 µm aperture to select the beam size, whereas images (*c*), (*d*), (*e*) and (*f*) use a 20 × 20 µm aperture to define the beam size. Owing to these differences in the setup, as well as the air path between the scatter guard and the entrance window and between the exit window and the beamstop, too much cannot be drawn from the differences in the background scatter. It can be seen, however, that when graphene/PMMA is present around the crystals and they are exposed to vacuum, diffraction remains until the end of data collection when compared with the data collection when the crystals are under vacuum but are not protected by graphene/PMMA.

Representative diffraction patterns of thaumatin within the vacuum chamber are shown in Fig. 3 ▸, with the corresponding data-collection and processing statistics in Table 1 ▸. Figs. 3 ▸(*a*) and 3 ▸(*b*) and Figs. 3 ▸(*c*) and 3 ▸(*d*) show the diffraction of thaumatin wrapped in graphene/PMMA in the chamber under air or vacuum, respectively. Under air reflections are visible to approximately 1.8 Å resolution and under vacuum reflections are visible to approximately 1.92 Å resolution. From these diffraction images there is a slight degradation in the diffracting power of the crystals wrapped in graphene/PMMA placed under vacuum, but no degradation in the quality of the reflections. This is also confirmed by the results in columns 2 and 3 of Table 1 ▸, where all graphene/PMMA-wrapped crystals produce reasonable data-quality statistics and consistent unit-cell parameters regardless of the atmosphere around them. Figs. 3 ▸(*e*) and 3 ▸(*f*) show diffraction from unwrapped thaumatin placed under vacuum. Although diffraction is still observed, the quality is visibly worse compared with those wrapped in graphene. The diffraction quality also drops off much more quickly in these crystals, with limited diffraction being visible in the last image of the data collection when compared with the same image for those crystals protected by graphene/PMMA. The results for this scenario are shown in column 4 of Table 1 ▸. The data extend to only 2.83 Å resolution and the values of *R*_meas_ are higher. There is a marked reduction in the unit-cell parameters, most notably in the *a* and *b* dimensions, which decrease from approximately 59 Å for wrapped crystals to 54 Å for unwrapped crystals exposed to vacuum.

**Figure 4:**
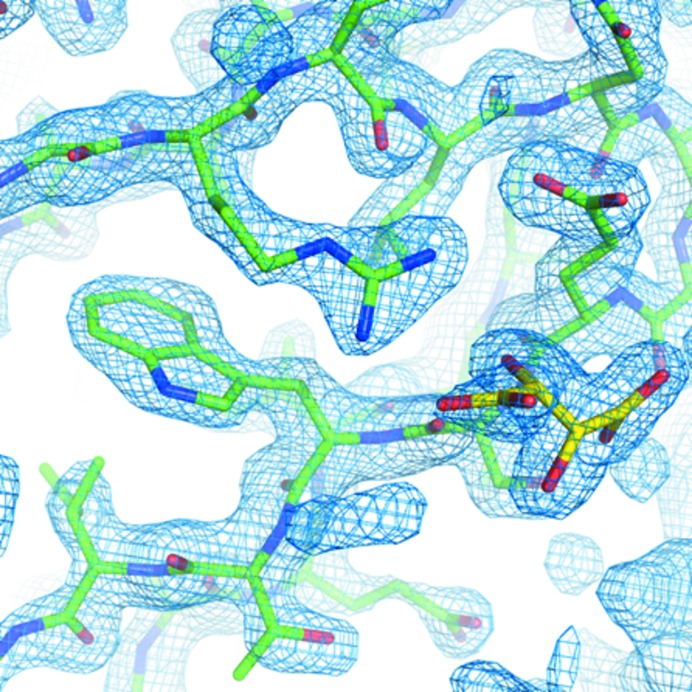
The 2*F*_o_ − *F*_c_ map shown at 1.5σ for the structure of thaumatin wrapped in graphene/PMMA exposed to vacuum. The overall thaumatin structure is shown in green and the tartrate ligand is shown in yellow, showing that it is possible to refine the ligand within the structure.

Table 2 ▸ and Fig. 4 ▸ show the structure-solution and refinement statistics and the electron-density map for thaumatin crystals wrapped in graphene/PMMA exposed to vacuum. The statistics demonstrate that relatively good figures of merit can be obtained for a room-temperature data collection under vacuum when protected by graphene/PMMA. The *B* factors from the refinement can be compared with a similar room-temperature data collection without graphene/PMMA and vacuum. For the wrapped thaumatin data collected here, the average atomic *B* factor is reported to be 38.57 Å^2^, while for an equivalent thaumatin room-temperature data set the average atomic *B* factor is 24.21 Å^2^ (unpublished work). This difference in *B* values might be attributable to the additional manual handling required during the wrapping process and may point to a potential problem with the current procedure.

**Figure 5:**
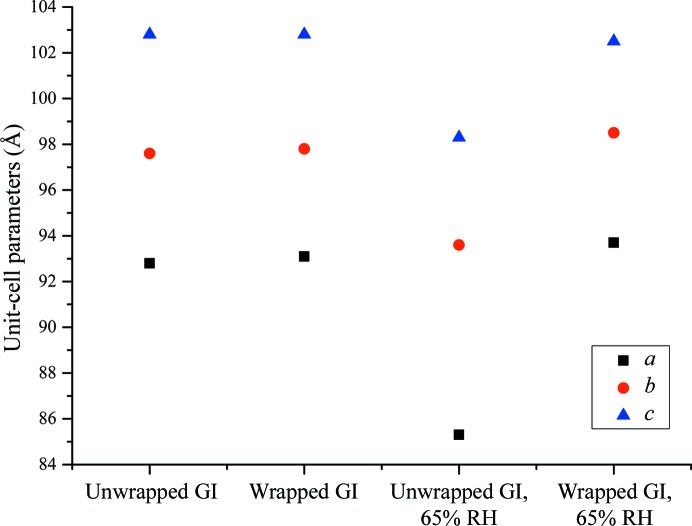
Plot of the unit-cell parameters from Table 3 ▸ illustrating how glucose isomerase (GI) crystals wrapped in graphene/PMMA are resistant to reduced humidity environments that would otherwise effect a significant unit-cell dimension contraction and, in this particular case, an associated space-group change from *I*222 to *P*2_1_2_1_2.

HC1b experiments were carried out on crystals of GI and the results of these data collections can be seen in Table 3 ▸ and Supplementary Tables S2–S5. Before any change in relative humidity is applied to the crystals the data quality is comparable between the crystals wrapped in graphene/PMMA and those that are unwrapped. When the relative humidity is decreased to 65%, the unwrapped crystals undergo a phase transition from *I*222 to *P*2_1_2_1_2. This is also accompanied by a decrease in the crystal quality, as observed by an increase in the value of *R*_meas_ and an increase in the *B* factor. As a comparison, GI crystals exposed to 65% relative humidity which are wrapped in graphene/PMMA show no phase transition, and the data quality remains consistent with the data sets where the change in relative humidity has not been applied. The changes in unit-cell parameters can be observed in Fig. 5 ▸ for all four conditions. The unit-cell axes are consistent for all data sets, except where the crystals are exposed to a relative humidity of 65% without protection by graphene/PMMA.

The results from the data collections carried out using the HC1b with lysozyme can be found in Table 4 ▸ and Supplementary Tables S6–S9. Without a change in relative humidity, both unwrapped and graphene/PMMA-wrapped crystals give comparable statistics. When 70% relative humidity is applied to a graphene/PMMA-wrapped crystal there is no significant change in crystal quality or the unit cell, as shown by the statistics in column 5 of Table 4 ▸. However, when the same relative humidity is applied to unwrapped crystals, data statistics such as the *R*_meas_ and *B*-factor values are much higher. In fact, in two out of the seven data sets treated with these conditions a reduction in the unit cell is also observed (Supplementary Table S8).

The data-processing statistics for the lysozyme crystal wrapped in graphene/PMMA and soaked in acetone can be seen in Table 5 ▸. There is a shrinkage in the unit-cell parameters, a slight increase in the *B* factor and a drop off in resolution when compared with a control data set in Table 4 ▸ (column 2).

## 4. Discussion   

The results outlined in this manuscript have demonstrated how effective multilayer graphene/PMMA can be at protecting protein crystals from dehydration once removed from their mother liquor. This was confirmed by several different methods. Although the PMMA was retained along with the graphene to aid in its manipulation, the permeability of PMMA to water has been well documented (Ellis & Smith, 2008 ▸; Salamone, 1996 ▸), indicating that graphene is principally responsible for providing the protection.

The first experiments outlined how graphene/PMMA can be used to protect crystals when exposed to vacuum without having to cryocool the crystals. The data sets where the crystals were wrapped in graphene/PMMA and then either exposed to vacuum or left in air were comparable in quality. When the crystals were left unwrapped and exposed to vacuum, the data quality was evidently worse. The unit-cell parameters in this case also showed a reduction, which is consistent with crystal dehydration. Surprisingly, however, these crystals still diffracted even under vacuum. This is probably explained by the retention of a small amount of mother liquor around the crystal during the mounting procedure that could act as a temporary barrier against dehydration. This was, however, not as effective as graphene/PMMA (see Table 1 ▸). This was supported by the diffraction images in Fig. 3 ▸, where the diffraction shown in Figs. 3 ▸(*e*) and 3 ▸(*f*) is degraded compared with that in Figs. 3(*a*), 3(*b*), 3(*c*) and 3(*d*), where the crystals are protected by graphene/PMMA.

The vacuum chamber provided a convenient method for testing samples under a rough vacuum; however, the chamber was not optimized for routine crystallographic data collection. The entrance and exit windows of the chamber limited the angular rotation to approximately 60°, but still allowed the collection of >95% complete data. The scatterguard and beamstop were also not optimally set up, with air gaps between these and the entrance and exit windows of the chamber. For this reason, analysis of the potential advantages of vacuum for reducing the background was not performed.

The results from graphene/PMMA-wrapped crystals in vacuum show relatively good data quality. However, the current process of mounting crystals in graphene/PMMA includes several manual handling steps that might be detrimental to the crystal and hence the data quality. We noticed that the quality of some data sets was reduced and refined atomic *B* factors were higher compared with similar structures determined from data collected using more standard means. This suggests that either the additional manual handling step is harming the crystals or that if the crystals were not completely sealed by the graphene/PMMA then slow dehydration might be taking place.

Tests carried out using the HC1b effectively demonstrate how graphene/PMMA can protect crystals from dehydration. For both the lysozyme and GI examples, there are significant changes in the lattice properties and data quality for unwrapped crystals exposed to lower relative humidity. The equivalent data sets for graphene/PMMA-wrapped crystals produced no such lattice changes or significant degradation in data quality. For lysozyme there is approximately a 3.5 Å reduction in the unit-cell axes, strongly suggesting dehydration of the crystal. For GI a phase transition from *I*222 to *P*2_1_2_1_2 is observed, which in this case is a known side effect of dehydration.

The results that we have presented support the claim that this method (Wierman *et al.*, 2013 ▸) of wrapping crystals in graphene or graphene/PMMA can protect crystals in an evacuated environment.

In all of these experiments the material used was multi-layer graphene coated with a 100 nm layer of PMMA. The PMMA acts as a support for the graphene, without which it is almost impossible to manipulate it for the purpose described here. We have demonstrated that multi-layer graphene/PMMA can be used as a covering to protect protein crystals. The samples studied here were on the scale of several tens of micrometres, making the 100 nm layer of PMMA negligible by comparison with respect to its impact on background scatter; however, the thickness of the PMMA could begin to play a role when working with micrometre-sized or smaller crystals.

Attempts were made to remove the PMMA from the graphene using a Kapton loop as a support. Here, a crystal of lysozyme was wrapped in graphene/PMMA and mounted on the Kapton loop. The mounted crystal in graphene/PMMA was soaked in acetone to remove the PMMA; however, some degradation in crystal quality was observed. The statistics in Table 5 ▸ are not as promising as those displayed in column 2 of Table 4 ▸. However, the action of soaking the sample in acetone is decidedly harsh, and it is surprising that the crystal still diffracts at all. The quality of this diffraction is still better than that of the dehydrated sample in column 4 of Table 4 ▸, indicating that some dehydration may have occurred. If the graphene was not completely sealed around the crystal then it may have been possible for a small amount of acetone to make contact with the crystal.

The experiments carried out here used relatively large crystals. For long-wavelength beamlines requiring *in vacuo* environments, a crystal size of <50 µm is typically used to limit absorption by the crystal itself (Liu *et al.*, 2013 ▸). It is likely that this methodology could easily transfer to crystals of a few tens of micrometres in size. However, it is less easy to imagine how graphene wrapping, as used here, is applicable to microcrystals or nanocrystals. Graphene or graphene/PMMA could act as an ideal low-scatter window or cover for microcrystal/nanocrystal preparations, but further investigation is required to develop this methodology.

## 5. Conclusion   

The results presented here demonstrate the use of graphene/PMMA as a layer to protect protein crystals from dehydration when exposed to vacuum. We have shown that it is possible to collect room-temperature data from protein crystals under vacuum, which has applicability in the development of new MX beamlines with in-vacuum sample environments. We have also confirmed these findings by collecting data from different samples at varying relative humidities and shown that no significant change in data quality is observed when the samples are wrapped in graphene/PMMA. Finally, we have shown that initial investigations into removal of the PMMA layer indicate that it may be feasible to use acetone post crystal-wrapping. Graphene has the potential to form a chemically resistant barrier for crystals, opening up possibilities for new types of experiments. It is interesting to consider that graphene/PMMA can create a sealed environment around the crystals that could be exploited as an anaerobic environment, for example.

With the recent renewed interest in the use of evacuated sample environments, it is timely to demonstrate a method for mounting and measuring data at room temperature from protein crystals in vacuum. The expectation is that significant gains in the signal-to-noise ratio from such data are possible now that the integrity of crystals can be maintained *in vacuo* using graphene/PMMA wraps that minimize the solvent surrounding the crystal.

## Supplementary Material

PDB reference: thaumatin wrapped in graphene, 4zxr

Supporting Information.. DOI: 10.1107/S1399004715014194/tz5077sup1.pdf

Video showing the mounting of crystals in graphene/PMMA.. DOI: 10.1107/S1399004715014194/tz5077sup2.mp4

## Figures and Tables

**Table 1:** Data-processing statistics of thaumatin crystals within the vacuum chamber A comparison between thaumatin wrapped in graphene/PMMA in air and vacuum and thaumatin in vacuum. Values in parentheses are for the outer shell.

	Graphene/PMMA-wrapped thaumatin in air	Graphene/PMMA-wrapped thaumatin under vacuum	Thaumatin under vacuum
Graphene/PMMA	X	X	
Vacuum		X	X
No. of images used in processing	485	485	485
Space group	*P*4_1_2_1_2	*P*4_1_2_1_2	*P*4_1_2_1_2
Unit-cell parameters ()	*a* = *b* = 59.1, *c* = 151.2	*a* = *b* = 59.1, *c* = 151.2	*a* = *b* = 53.9, *c* = 152.1
Resolution range ()	46.571.80 (1.841.80)	41.781.92 (1.971.92)	50.792.83 (2.902.83)
Total No. of reflections	86945 (5233)	73107 (4986)	19170 (2741)
No. of unique reflections	24715 (1467)	20125 (1390)	5790 (800)
Completeness (%)	97.0 (98.4)	95.9 (98.8)	99.5 (98.7)
Multiplicity	3.5 (3.6)	3.6 (3.6)	3.3 (3.5)
*I*/(*I*)	9.6 (2.2)	10.2 (2.0)	7.3 (2.2)
*R*_meas_	0.080 (0.763)	0.068 (0.676)	0.153 (0.699)
Overall *B* factor from Wilson plot† (^2^)	29.1	31.4	65.9

† Calculated with the *WILSON* program in the *CCP*4 suite (Winn *et al.*, 2011 ▸).

**Table 2:** Thaumatin refinement statistics against data from graphene/PMMA-wrapped crystals collected under vacuum Values in parentheses are for the outer shell.

Resolution range ()	41.781.92 (1.971.92)
Completeness (%)	95.9 (98.8)
No. of reflections, working set	19143
No. of reflections, test set	982
Final *R*_cryst_	0.146
Final *R*_free_	0.175
No. of non-H atoms	
Protein	1526
Ligand	22
Water	87
Total	1635
R.m.s. deviations	
Bonds ()	0.0222
Angles ()	2.1822
Average *B* factors (^2^)	
Overall	38.57
Protein	37.84
Ligand	46.78
Water	49.32
Ramachandran plot	
Most favoured (%)	98.5
Allowed (%)	1.5

**Table 3:** Data-processing statistics of GI crystals studied with the HC1b A comparison between the controls, GI and GI wrapped in graphene/PMMA, with an unwrapped GI crystal and a GI crystal wrapped in graphene/PMMA exposed to a relative humidity of 65%. Although only one data set is shown for each sample, at least three crystals were collected for each scenario; the statistics from all of the crystals can be found in the Supporting Information. All data sets showed a similar trend for each scenario. Values in parentheses are for the outer shell.

	GI	Graphene/PMMA-wrapped GI	GI exposed to 65% RH	Graphene/PMMA-wrapped GI exposed to 65% RH
Graphene/PMMA		X		X
Relative humidity applied at 65%			X	X
Space group	*I*222	*I*222	*P*2_1_2_1_2	*I*222
Unit-cell parameters ()	*a* = 92.8, *b* = 97.6, *c* = 102.8	*a* = 93.1, *b* = 97.8, *c* = 102.8	*a* = 85.3, *b* = 93.6, *c* = 98.3	*a* = 93.7, *b* = 98.5, *c* = 102.5
Resolution range ()	29.631.80 (1.851.80)	67.441.80 (1.851.80)	32.761.87 (1.911.87)	69.141.80 (1.851.80)
Total No. of reflections	274203 (19683)	276968 (20925)	417412 (26430)	291841 (21602)
No. of unique reflections	43300 (3099)	43701 (3216)	65681 (4206)	44175 (3229)
Completeness (%)	99.7 (97.2)	99.9 (99.9)	100 (100)	99.9 (99.9)
Multiplicity	6.3 (6.4)	6.3 (6.5)	6.4 (6.3)	6.6 (6.7)
*I*/(*I*)	26.4 (11.8)	32.4 (18.4)	9.4 (2.2)	28.7 (12.8)
*R*_meas_	0.049 (0.132)	0.041 (0.078)	0.288 (0.753)	0.062 (0.273)
Overall *B* factor from Wilson plot (^2^)	12.4	10.5	15.7	7.7

**Table 4:** Data-processing statistics of lysozyme crystals studied with the HC1b A comparison between the controls, lysozyme and lysozyme wrapped in graphene/PMMA, with an unwrapped lysozyme crystal and a lysozyme crystal wrapped in graphene/PMMA exposed to a relative humidity of 70%. Although only one data set is shown for each sample, at least three crystals were collected for each scenario; the statistics from all of the crystals can be found in the Supporting Information. All data sets showed a similar trend for each scenario, except in the case where a relative humidity of 70% was applied to an unwrapped crystal. In this case seven data sets were collected, all of which showed an increase in the values of *R*_meas_ and the *B* factor, and in two of the seven data sets a reduction in the unit cell was also observed. Values in parentheses are for the outer shell.

	Lysozyme	Graphene/PMMA-wrapped lysozyme	Lysozyme exposed to 70% RH	Graphene/PMMA-wrapped lysozyme exposed to 70% RH
Graphene/PMMA		X		X
Relative humidity applied at 70%			X	X
Space group	*P*4_3_2_1_2	*P*4_3_2_1_2	*P*4_3_2_1_2	*P*4_3_2_1_2
Unit-cell parameters ()	*a* = *b* = 78.0, *c* = 37.1	*a* = *b* = 77.8, *c* = 38.0	*a* = *b* = 74.3, *c* = 33.6	*a* = *b* = 78.1, *c* = 37.4
Resolution range ()	34.891.70 (1.741.70)	34.141.70 (1.751.70)	30.642.84 (2.992.84)	33.701.70 (1.741.70)
Total No. of reflections	157599 (9480)	157565 (9084)	28043 (4185)	160618 (9573)
No. of unique reflections	13018 (908)	13281 (946)	2475 (347)	13233 (942)
Completeness (%)	99.4 (96.8)	99.9 (99.3)	99.9 (100)	99.9 (99.8)
Multiplicity	12.1 (10.4)	11.9 (9.6)	11.3 (12.1)	12.1 (10.2)
*I*/(*I*)	45.1 (17.9)	48.5 (29.6)	18.3 (2.0)	46.3 (14.2)
*R*_meas_	0.040 (0.104)	0.048 (0.065)	0.076 (1.427)	0.034 (0.137)
Overall *B* factor from Wilson plot (^2^)	14.0	14.4	109.9	18.4

**Table 5:** Data-processing statistics of lysozyme crystals when wrapped in graphene/PMMA and soaked in acetone These statistics can be compared with those in Table 4 ▸.

Space group	*P*4_3_2_1_2
Unit-cell parameters ()	*a* = *b* = 76.9, *c* = 36.9
Resolution range ()	25.622.27 (2.332.27)
Total No. of reflections	56525 (4635)
No. of unique reflections	5095 (403)
Completeness (%)	93.1 (99.9)
Multiplicity	11.1 (11.5)
*I*/(*I*)	10.2 (2.2)
*R*_meas_	0.150 (0.974)
Overall *B* factor from Wilson plot (^2^)	99.0

## References

[bb1] Axford, D. *et al.* (2012). *Acta Cryst.* D**68**, 592–600. 10.1107/S0907444912006749 PMC4791750 22525757

[bb2] Bingel-Erlenmeyer, R., Olieric, V., Grimshaw, J. P. A., Gabadinho, J., Wang, X., Ebner, S. G., Isenegger, A., Schneider, R., Schneider, J., Glettig, W., Pradervand, C., Panepucci, E. H., Tomizaki, T., Wang, M. & Schulze-Briese, C. (2011). *Cryst. Growth Des.* **11**, 916–923.

[bb3] Boutet, S. & Williams, G. J. (2010). *New J. Phys.* **12**, 035024.

[bb4] Bunch, J. S., Verbridge, S. S., Alden, J. S., van der Zande, A. M., Parpia, J. M., Craighead, H. G. & McEuen, P. L. (2008). *Nano Lett.* **8**, 2458–2462. 10.1021/nl801457b 18630972

[bb5] Chapman, H. N. *et al.* (2011). *Nature (London)*, **470**, 73–77.

[bb6] Djinović Carugo, K., Helliwell, J. R., Stuhrmann, H. & Weiss, M. S. (2005). *J. Synchrotron Rad.* **12**, 410–419. 10.1107/S0909049504025762 15968116

[bb7] Dobrianov, I., Kriminski, S., Caylor, C. L., Lemay, S. G., Kimmer, C., Kisselev, A., Finkelstein, K. D. & Thorne, R. E. (2001). *Acta Cryst.* D**57**, 61–68. 10.1107/s0907444900014578 11134928

[bb8] Duke, E. M. H. & Johnson, L. N. (2010). *Proc. R. Soc. A*, **466**, 3421–3452.

[bb9] Duran, D., Couster, S. L., Desjardins, K., Delmotte, A., Fox, G., Meijers, R., Moreno, T., Savko, M. & Shepard, W. (2013). *J. Phys. Conf. Ser.* **425**, 012005.

[bb35] Ellis, B. & Smith, R. (2008). Editors. *Polymers: A Property Database*, 2nd ed. Boca Raton: CRC Press.

[bb10] Emsley, P., Lohkamp, B., Scott, W. G. & Cowtan, K. (2010). *Acta Cryst.* D**66**, 486–501. 10.1107/S0907444910007493 PMC2852313 20383002

[bb11] Evans, G., Axford, D. & Owen, R. L. (2011). *Acta Cryst.* D**67**, 261–270. 10.1107/S0907444911007608 PMC3069741 21460444

[bb12] Evans, G., Axford, D., Waterman, D. & Owen, R. L. (2011). *Crystallogr. Rev.* **17**, 105–142.

[bb13] Evans, P. R. & Murshudov, G. N. (2013). *Acta Cryst.* D**69**, 1204–1214. 10.1107/S0907444913000061 PMC3689523 23793146

[bb14] Fischetti, R. F., Yoder, D., Xu, S., Makarov, O., Ogata, C. & Smith, J. L. (2013). *J. Phys. Conf. Ser.* **425**, 012006. 10.1088/1742-6596/425/1/012006 PMC4222077 25383086

[bb15] Glaeser, R., Facciotti, M., Walian, P., Rouhani, S., Holton, J., MacDowell, A., Celestre, R., Cambie, D. & Padmore, H. (2000). *Biophys. J.* **78**, 3178–3185. 10.1016/S0006-3495(00)76854-8 PMC1300899 10827994

[bb16] Hendrickson, W. A. & Ogata, C. M. (1997). *Methods Enzymol.* **276**, 494–523. 10.1016/S0076-6879(97)76074-9 27799111

[bb17] Hirata, K., Kawano, Y., Ueno, G., Hashimoto, K., Murakami, H., Hasegawa, K., Hikima, T., Kumasaka, T. & Yamamoto, M. (2013). *J. Phys. Conf. Ser.* **425**, 012002.

[bb18] Holton, J. M. & Frankel, K. A. (2010). *Acta Cryst.* D**66**, 393–408. 10.1107/S0907444910007262 PMC2852304 20382993

[bb19] Kabsch, W. (2010). *Acta Cryst.* D**66**, 125–132. 10.1107/S0907444909047337 PMC2815665 20124692

[bb20] Krieger, M. & Stroud, R. M. (1976). *Acta Cryst.* A**32**, 653–656.

[bb21] Lehmann, M. S., Müller, H.-H. & Stuhrmann, H. B. (1993). *Acta Cryst.* D**49**, 308–310. 10.1107/S0907444992011910 15299536

[bb22] Lin, J.-B., Xue, W., Zhang, J.-P. & Chen, X.-M. (2011). *Chem. Commun.* **47**, 926–928. 10.1039/c0cc04089d 21076763

[bb23] Liu, Q., Liu, Q. & Hendrickson, W. A. (2013). *Acta Cryst.* D**69**, 1314–1332. 10.1107/S0907444913001479 PMC3689535 23793158

[bb24] Liu, Y., Ogata, C. M. & Hendrickson, W. A. (2001). *Proc. Natl Acad. Sci. USA*, **98**, 10648–10653. 10.1073/pnas.191003998 PMC58520 11526210

[bb25] Lobley, C. M. C., Sandy, D. J., Sanchez-Weatherby, J., Mazzorana, M., Krojer, T., Nowak, R. P. & Sorensen, T. L. (2015). Submitted. 10.1107/S2059798316003065 PMC4854313 27139626

[bb26] Madsen, D., Burghammer, M., Fiedler, S. & Müller, H. (1999). *Acta Cryst.* B**55**, 601–606. 10.1107/s0108768199003468 10927401

[bb27] Mazzorana, M., Sanchez-Weatherby, J., Sandy, J., Lobley, C. M. C. & Sorensen, T. (2014). *Acta Cryst.* D**70**, 2390–2400. 10.1107/S1399004714014370 PMC4157448 25195752

[bb28] Mueller, M., Wang, M. & Schulze-Briese, C. (2012). *Acta Cryst.* D**68**, 42–56. 10.1107/S0907444911049833 PMC3245722 22194332

[bb29] Murshudov, G. N., Skubák, P., Lebedev, A. A., Pannu, N. S., Steiner, R. A., Nicholls, R. A., Winn, M. D., Long, F. & Vagin, A. A. (2011). *Acta Cryst.* D**67**, 355–367. 10.1107/S0907444911001314 PMC3069751 21460454

[bb30] Mykhaylyk, V. & Wagner, A. (2013). *J. Phys. Conf. Ser.* **425**, 012010.

[bb31] Nannenga, B. L., Shi, D., Leslie, A. G. W. & Gonen, T. (2014). *Nature Methods*, **11**, 927–930. 10.1038/nmeth.3043 PMC4149488 25086503

[bb32] Owen, R. L., Paterson, N., Axford, D., Aishima, J., Schulze-Briese, C., Ren, J., Fry, E. E., Stuart, D. I. & Evans, G. (2014). *Acta Cryst.* D**70**, 1248–1256. 10.1107/S1399004714005379 PMC4014120 24816094

[bb33] Perutz, M. F. & Rogers, G. L. (1946). *J. Sci. Instrum.* **23**, 217.

[bb34] Salamone, J. C. (1996). Editor. *Polymeric Materials Encyclopedia*, Vol. 1. Boca Raton: CRC Press.

[bb36] Sanchez-Weatherby, J., Bowler, M. W., Huet, J., Gobbo, A., Felisaz, F., Lavault, B., Moya, R., Kadlec, J., Ravelli, R. B. G. & Cipriani, F. (2009). *Acta Cryst.* D**65**, 1237–1246. 10.1107/S0907444909037822 19966409

[bb37] Sauter, C., Lorber, B. & Giegé, R. (2002). *Proteins*, **48**, 146–150. 10.1002/prot.10125 12112683

[bb38] Schneider, D. K., Berman, L. E., Chubar, O., Hendrickson, W. A., Hulbert, S. L., Lucas, M., Sweet, R. M. & Yang, L. (2013). *J. Phys. Conf. Ser.* **425**, 012003.

[bb39] Smith, J. L., Fischetti, R. F. & Yamamoto, M. (2012). *Curr. Opin. Struct. Biol.* **22**, 602–612. 10.1016/j.sbi.2012.09.001 PMC3478446 23021872

[bb40] Suk, J. W., Kitt, A., Magnuson, C. W., Hao, Y., Ahmed, S., An, J., Swan, A. K., Goldberg, B. B. & Ruoff, R. S. (2011). *ACS Nano*, **5**, 6916–6924. 10.1021/nn201207c 21894965

[bb41] Thorne, R. E., Stum, Z., Kmetko, J., O’Neill, K. & Gillilan, R. (2003). *J. Appl. Cryst.* **36**, 1455–1460.

[bb42] Weierstall, U., Spence, J. C. H. & Doak, R. B. (2012). *Rev. Sci. Instrum.* **83**, 035108. 10.1063/1.3693040 22462961

[bb43] Wierman, J. L., Alden, J. S., Kim, C. U., McEuen, P. L. & Gruner, S. M. (2013). *J. Appl. Cryst.* **46**, 1501–1507. 10.1107/S002188981301786X PMC3778323 24068843

[bb45] Winn, M. D. *et al.* (2011). *Acta Cryst.* D**67**, 235–242.

[bb44] Winter, G., Lobley, C. M. C. & Prince, S. M. (2013). *Acta Cryst.* D**69**, 1260–1273. 10.1107/S0907444913015308 PMC3689529 23793152

