# Supplementary material for: In vacuo X-ray data collection from graphene-wrapped protein crystals
Source: Acta Crystallogr D Biol Crystallogr. 2015 Sep 26;71(Pt 10):2079–88. doi: 10.1107/S1399004715014194 (PMC4601369; doi:10.1107/S1399004715014194)
Supplement: Supplementary file 1 [file d-71-02079-sup1.pdf]

BIOLOGICAL CRYSTALLOGRAPHY

Volume 71 (2015)

Supporting information for article:

In vacuo X-ray data collection from graphene-wrapped protein crystals

Anna J. Warren, Adam D. Crawshaw, Jose Trincao, Pierre Aller, Simon Alcock, Ioana Nistea, Paula S. Salgado and Gwyndaf Evans

S1. Measurement of PMMA thickness on graphene/PMMA

The graphene used in this investigation was Trivial Transfer graphene purchased from ACS Materials. It is supplied as 3-5 layers of graphene spin coated with PMMA (poly(methyl methacrylate)), with a quoted PMMA thickness of ~500 nm (acsmaterial.com). The thickness of the PMMA was measured by micro-interferometry on a Bruker Contour GT-X and found to be 100 +/- 10 nm (Figure S1).

S2. Confirmation of PMMA removal from graphene

The PMMA layer can be removed from the graphene/PMMA with the use of acetone. The graphene/PMMA should be free of any excess water and should be left to completely dry. After this a few drops of acetone can be applied to the surface of the graphene/PMMA ensuring that this surface is completely covered. The removal of the PMMA layer was confirmed with Infrared spectroscopy and micro-interferometry. From the interferometry results in Figure S2 it can be seen the total thickness is approximately 6 +/- 2 nm, indicating that the majority of the PMMA has been removed from the surface of the multi-layer graphene. Figure S3 shows the Infrared spectroscopy results, it is evident from these spectra that PMMA is present in the as-supplied Trivial Transfer graphene, but has been removed on treatment with acetone.

S3. Mounting of crystals in graphene/PMMA

The method reported here was adapted from that reported by Wierman *et al*. (Wierman *et al.*, 2013) but is broadly similar. A custom loop was 3D-printed to aid with the mounting of crystals in graphene/PMMA (Figure S4). The large custom loop was filled with the crystallisation reservoir solution using a pipette and visually checked to ensure the top and bottom surfaces had a low curvature. This allowed for easier manipulation of the graphene/PMMA on the reservoir solution. A small piece of graphene/PMMA was torn from a larger section using an acupuncture needle and then transferred to the top of the reservoir solution. This ripping action of the graphene/PMMA usually led to uneven shaped/sized sheets. Once on the top of the liquid, the loop was rotated 180 degrees so the graphene/PMMA was now on the bottom of the liquid. At this point crystals were pipetted into the drop above the graphene/PMMA and gently manipulated with an acupuncture needle towards the surface of the graphene/PMMA. A nylon or Kapton loop was then used to mount the crystal by bringing the loop, from the side, into the bottom of the drop and sweeping the loop down through the crystal and the graphene/PMMA sheet. This downward motion leaves the crystal and loop wrapped in the graphene/PMMA having pushed out the solution within the loop. The mounting procedure is shown in a video as part of the supplementary material.

S4. Resistance of graphene/PMMA-wrapped crystals to vacuum

The data merging statistics for the thaumatin crystals within the vacuum chamber are shown in Table S1.

S5. Resistance of graphene/PMMA-wrapped crystals to dehydration

All the data processing statistics for the GI and lysozyme crystals studied with the hc1b can be found in Tables S2, S3, S4 and S5 and Tables S6, S7, S8 and S9 respectively.

Table S1 Data processing statistics of thaumatin crystals within the vacuum chamber. A comparison between thaumatin wrapped in graphene/PMMA in air and vacuum, and thaumatin in vacuum.

	Graphene/PMMA-wrapped thaumatin in air	Graphene/PMMA-wrapped thaumatin under vacuum	Graphene/PMMA-wrapped thaumatin under vacuum	Graphene/PMMA-wrapped thaumatin under vacuum	Thaumatin under vacuum
Graphene/PMMA	X	X	X	X	
Vacuum		X	X	X	X
No. images used in processing	485	485	200	200	485
Space group	$P4_12_12$	$P4_12_12$	$P4_12_12$	$P4_12_12$	$P4_12_12$
a=*b*, *c* (Å)	59.1, 151.2	59.1, 151.2	59.0, 151.3	59.0, 151.3	53.9, 152.1
Resolution range (Å)	46.57-1.80(1.84-1.80)	41.78-1.92(1.97-1.92)	41.75-2.08(2.14-2.08)	46.63-2.98(3.13-2.95)	50.79-2.83(2.90-2.83)
Total no. of reflections	86945(5233)	73107(4986)	23912(1869)	8467(1408)	19170(2741)
No. of unique reflections	24715(1467)	20125(1390)	12579(1042)	4190(695)	5790(800)
Completeness (%)	97.0(98.4)	95.9(98.8)	77.2(82.8)	69.7(72.4)	99.5(98.7)
Redundancy	3.5(3.6)	3.6(3.6)	1.9(1.8)	2.0(2.0)	3.3(3.5)
$\langle I/\sigma(I) \rangle$	9.6(2.2)	10.2(2.0)	6.2(2.1)	2.2(2.0)	7.3(2.2)
R_{meas}	0.080(0.763)	0.068(0.676)	0.091(0.484)	0.051(0.077)	0.153(0.699)
Overall *B* factor from Wilson plot (Å^2)*	29.1	31.4	31.1	45.7	65.9

(*) Calculated with Wilson program in CCP4 suite (ref: Overview of the CCP4 suite and current developments". Acta Cryst. D67, 235-242.)

Table S2 Data processing statistics for the GI control where crystals were immediately cryo-cooled in liquid nitrogen for data collection.

	GI	GI	GI	GI
Space group	*I*222	*I*222	*I*222	*I*222
a, *b*, *c* (Å)	92.8, 97.5, 102.9	92.8, 97.6, 102.8	92.9, 97.7, 102.7	92.9, 97.3, 102.6
Resolution range (Å)	26.53-1.80(1.85-1.80)	29.63-1.80(1.85-1.80)	31.05-1.80(1.85-1.80)	29.65-1.80(1.85-1.80)
Total no. of reflections	271556(20447)	274203(19683)	283436(20140)	276957(20428)
No. of unique reflections	43420(3170)	43300 (3099)	43519(3141)	43293 (3137)
Completeness (%)	99.9(99.9)	99.7(97.2)	99.8(98.6)	99.9(99.9)
Redundancy	6.3(6.5)	6.3(6.4)	6.5(6.4)	6.4(6.5)
$\langle I/\sigma(I)\rangle$	31.2(16.5)	26.4(11.8)	19.8(7.2)	24.8(8.7)
R_{meas}	0.042(0.089)	0.049(0.132)	0.071(0.266)	0.056(0.219)
Overall *B* factor from Wilson plot (Å^2)	10.9	12.4	11.9	12.6

Table S3 Data processing statistics for the GI wrapped in graphene/PMMA control where crystals were wrapped in graphene and immediately cryo-cooled in liquid nitrogen for data collection.

	Graphene/PMMA-wrapped GI	Graphene/PMMA-wrapped GI	Graphene/PMMA-wrapped GI
Space group	*I*222	*I*222	*I*222
a, *b*, *c* (Å)	93.1, 97.9, 102.8	93.1, 97.8, 102.8	93.1, 98.1, 102.7
Resolution range (Å)	68.99-1.80(1.85-1.80)	67.44-1.80(1.85-1.80)	70.93-1.80(1.85-1.80)
Total no. of reflections	279707(21171)	276968(20925)	284699(20869)
No. of unique reflections	43730(3201)	43701 (3216)	43733(3191)
Completeness (%)	99.9(99.8)	99.9(99.9)	99.8(98.8)
Redundancy	6.4(6.6)	6.3(6.5)	6.5(6.5)
$\langle I/\sigma(I)\rangle$	40.9(23.0)	32.4(18.4)	25.7(12.8)
R_{meas}	0.033(0.062)	0.041(0.078)	0.059(0.188)
Overall *B* factor from Wilson plot (Å^2)	10.5	10.5	8.6

Table S4 Data processing statistics for the GI wrapped when exposed to 65% relative humidity for 5 minutes before crystals were cryocooled in liquid nitrogen for data collection.

	GI exposed to RH 65%	GI exposed to RH 65%	GI exposed to RH 65%
Space group	$P2_12_12$	$P2_12_12$	$P2_12_12$
a, *b*, *c* (Å)	84.9, 93.1, 97.9	85.3, 93.6, 98.3	82.6, 94.0, 97.6
Resolution range (Å)	29.87-1.94(1.99-1.94)	32.76-1.87(1.91-1.87)	31.54-1.80(1.85-1.80)
Total no. of reflections	363361(25556)	417412(26430)	444604(32762)
No. of unique reflections	58101(3858)	65681(4206)	70459 (5177)
Completeness (%)	99.9(100)	100(100)	99.2(99.8)
Redundancy	6.3(6.6)	6.4(6.3)	6.3(6.3)
$\langle I/\sigma(I)\rangle$	5.2(2.1)	9.4(2.2)	10.4(2.0)
R_{meas}	0.288(0.753)	0.288(0.753)	0.172(0.857)
Overall *B* factor from Wilson plot (Å^2)	15.7	18.8	24.8

Table S5 Data processing statistics for the GI crystals wrapped in graphene/PMMA then exposed to 65% relative humidity for 5 minutes before crystals were cryo-cooled in liquid nitrogen for data collection.

	Graphene/PMMA-wrapped GI exposed to RH 65%	Graphene/PMMA-wrapped GI exposed to RH 65%	Graphene/PMMA-wrapped GI exposed to RH 65%	Graphene/PMMA-wrapped GI exposed to RH 65%
Space group	*I*222	*I*222	*I*222	*I*222
a, *b*, *c* (Å)	93.7, 98.5, 102.5	93.6, 98.1, 102.4	93.8, 98.9, 102.3	93.8, 98.7, 102.5
Resolution range (Å)	69.14-1.80(1.85-1.80)	69.08-1.80(1.85-1.80)	40.89-1.80(1.85-1.80)	71.09-1.80(1.85-1.80)
Total no. of reflections	291841(21602)	283945(20919)	280209(19570)	286069(20046)
No. of unique reflections	44175(3229)	43849(3186)	43842(3109)	44336(3248)
Completeness (%)	99.9(99.9)	99.8(98.3)	99.7(96.8)	99.9(99.8)
Redundancy	6.6(6.7)	6.5(6.6)	6.5(6.3)	6.5(6.2)
$\langle I/\sigma(I)\rangle$	28.7(12.8)	32.8(14.2)	20.0(6.4)	15.8(5.0)
R_{meas}	0.062(0.273)	0.040(0.129)	0.063(0.310)	0.088(0.432)
Overall *B* factor from Wilson plot ($Å^2$)	7.7	12.4	17.7	14.1

Table S6 Data processing statistics for the lysozyme control where crystals were immediately cryo-cooled in liquid nitrogen for data collection.

	Lysozyme	Lysozyme	Lysozyme
Space group	$P4_32_12$	$P4_32_12$	$P4_32_12$
a=*b*, *c* (Å)	77.6, 36.9	77.8, 37.0	78.1, 37.1
Resolution range (Å)	33.35-1.70(1.75-1.70)	37.00-1.70(1.74-1.70)	34.89-1.70(1.74-1.70)
Total no. of reflections	153134(9181)	158612(9565)	157599(9480)
No. of unique reflections	12899(918)	13027(930)	13018(908)
Completeness (%)	100(99.8)	99.9(99.3)	99.4(96.8)
Redundancy	11.9(10.0)	12.2(10.3)	12.1(10.4)
$\langle I/\sigma(I)\rangle$	24.1(3.5)	48.9(24.0)	45.1(17.9)
R_{meas}	0.060(0.664)	0.039(0.075)	0.040(0.104)
Overall *B* factor from Wilson plot (Å^2)	22.2	16.5	14.0

Table S7 Data processing statistics for the lysozyme wrapped in graphene/PMMA control where crystals were wrapped in graphene immediately cryo-cooled in liquid nitrogen for data collection.

	Graphene/PMMA-wrapped lysozyme	Graphene/PMMA-wrapped lysozyme	Graphene/PMMA-wrapped lysozyme
Space group	$P4_32_12$	$P4_32_12$	$P4_32_12$
$a=b$, c (Å)	77.7, 38.2	77.8, 38.0	77.8, 38.0
Resolution range (Å)	34.76-1.70(1.75-1.70)	34.14-1.70(1.75-1.70)	34.79-1.70(1.74-1.70)
Total no. of reflections	166819(10113)	157565(9084)	163532(9948)
No. of unique reflections	13384(969)	13281(946)	13362(961)
Completeness (%)	100(100)	99.9(99.3)	100(99.9)
Redundancy	12.5(10.4)	11.9(9.6)	12.2(10.4)
$\langle I/\sigma(I)\rangle$	34.4(7.0)	48.5(29.6)	59.3(23.7)
R_{meas}	0.054(0.359)	0.048(0.065)	0.030(0.085)
Overall B factor from Wilson plot (Å^2)	14.0	14.4	14.0

Table S8 Data processing statistics for the lysozyme crystals exposed to 70% relative humidity for 5 minutes before crystals were cryo-cooled in liquid nitrogen for data collection.

	Lysozyme exposed to RH 70%	Lysozyme exposed to RH 70%	Lysozyme exposed to RH 70%	Lysozyme exposed to RH 70%
Space group	$P4_32_12$	$P4_32_12$	$P4_32_12$	$P4_32_12$
a=b, c (Å)	74.5, 34.1	77.5, 37.0	74.3, 33.6	77.8, 37.9
Resolution range (Å)	37.25-3.55(3.89-3.55)	38.77-1.77(1.81-1.77)	30.64-2.84(2.99-2.84)	38.92-2.21(2.28-2.21)
Total no. of reflections	12992(3000)	137940(7270)	28043(4185)	70770(5951)
No. of unique reflections	1328(302)	11508(928)	2475(347)	6225(520)
Completeness (%)	99.7(99.6)	100(100)	99.9(100)	100(100)
Redundancy	9.8(9.9)	12.0(11.6)	11.3(12.1)	11.4(11.4)
⟨$I/\sigma(I)$⟩	14.2(2.0)	19.1(2.1)	18.3(2.0)	9.4(2.1)
R_{meas}	0.080(1.048)	0.072(1.129)	0.076(1.427)	0.157(1.159)
Overall B factor from Wilson plot (Å^2)	132.9	14.0	109.9	48.9

	Lysozyme exposed to RH 70%	Lysozyme exposed to RH 70%	Lysozyme exposed to RH 70%
Space group	$P4_32_12$	$P4_32_12$	$P4_32_12$
a=b, c (Å)	77.0, 37.2	77.7, 37.3	77.6, 37.7
Resolution range (Å)	34.44-2.13(2.19-2.13)	34.75-1.70(1.73-1.70)	34.72-1.90(1.94-1.90)
Total no. of reflections	67632(4626)	140243(4526)	107431(6247)
No. of unique reflections	6617(510)	13064(603)	9545(583)
Completeness (%)	99.2(94.7)	99.0(89.2)	99.8(100)
Redundancy	10.2(9.1)	10.7(7.5)	11.3(10.7)
⟨$I/\sigma(I)$⟩	10.7(2.2)	16.7(2.3)	14.3(2.1)
R_{meas}	0.126(0.819)	0.084(0.829)	0.117(1.288)
Overall B factor from Wilson plot (Å^2)	34.6	21.2	26.4

Table S9 Data processing statistics for the lysozyme crystals wrapped in graphene/PMMA then exposed to 70% relative humidity for 5 minutes before crystals were cryo-cooled in liquid nitrogen for data collection.

	Graphene/PMMA-wrapped lysozyme exposed to RH 70%	Graphene/PMMA-wrapped lysozyme exposed to RH 70%	Graphene/PMMA-wrapped lysozyme exposed to RH 70%	Graphene/PMMA-wrapped lysozyme exposed to RH 70%
Space group	$P4_32_12$	$P4_32_12$	$P4_32_12$	$P4_32_12$
a=b, c (Å)	78.4, 37.3	78.7, 37.28	78.1, 37.35	78.1, 37.43
Resolution range (Å)	33.70-1.70(1.74-1.70)	39.33-1.70(1.74-1.70)	33.70-1.70(1.74-1.70)	33.75-1.70(1.74-1.70)
Total no. of reflections	162232(9757)	163572(9645)	160618(9573)	162084(9426)
No. of unique reflections	13328(953)	13417(963)	13233(942)	13247(923)
Completeness (%)	100(99.7)	100(99.8)	99.9(99.8)	99.8(98.2)
Redundancy	12.2(10.2)	12.2(10.0)	12.1(10.2)	12.2(10.2)
$\langle I/\sigma(I)\rangle$	56.2(20.3)	36.5(8.5)	46.3(14.2)	37.3(11.6)
R_{meas}	0.030(0.096)	0.051(0.28)	0.034(0.137)	0.051(0.239)
Overall B factor from Wilson plot (Å^2)	15.1	13.4	18.4	11.6

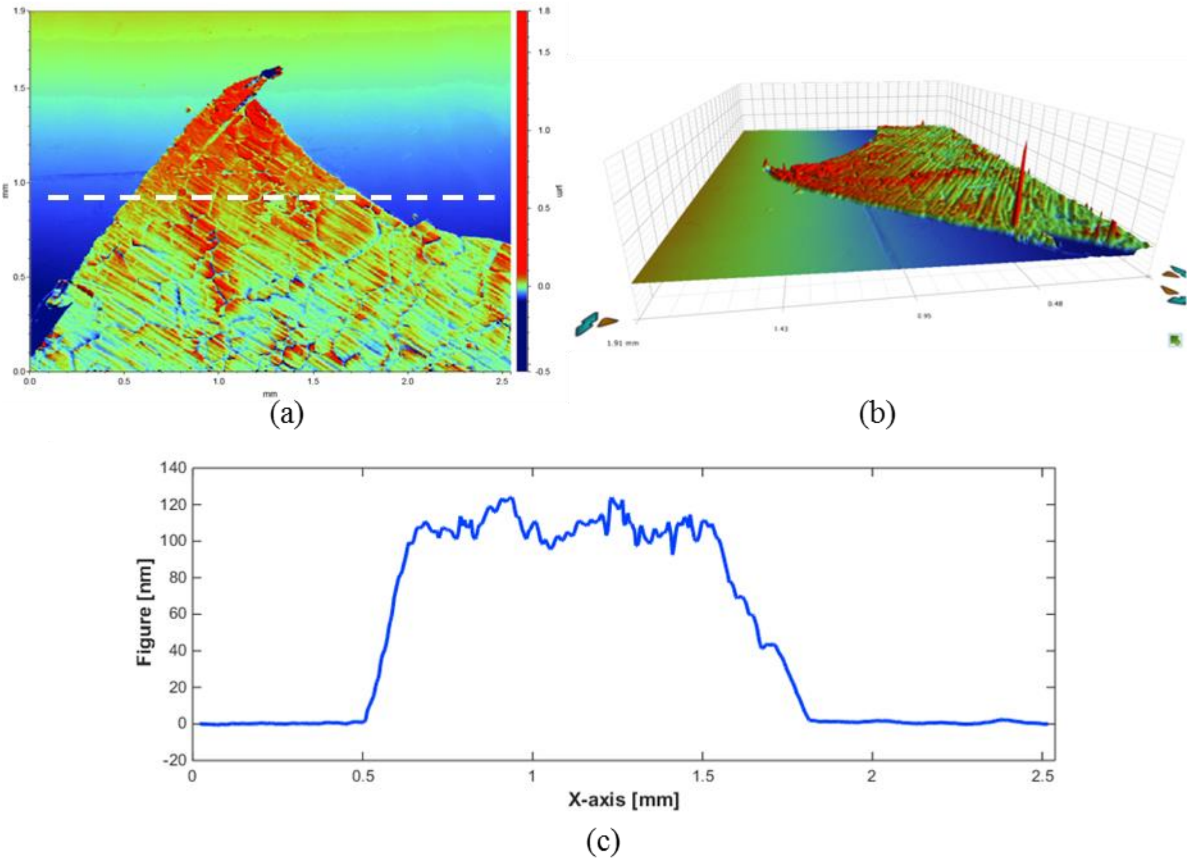

Figure S1 Micro-interferometry of the as-supplied Trivial Transfer graphene before removal of PMMA. (a) and (b) show 2D and 3D topography of the graphene/PMMA sample. The colours represent the scale of thickness from approximately 1.8 µm (red) to -0.5 µm (blue) (the point at which the zero level was specified is arbitrary, and the 1.8 µm peak height refers to a spike in the data as observed in (b)). A representative cross section, indicated in image (a) by the white line, is shown in (c). The thickness of the combined graphene/PMMA layer is approximately 100 +/- 10 nm.

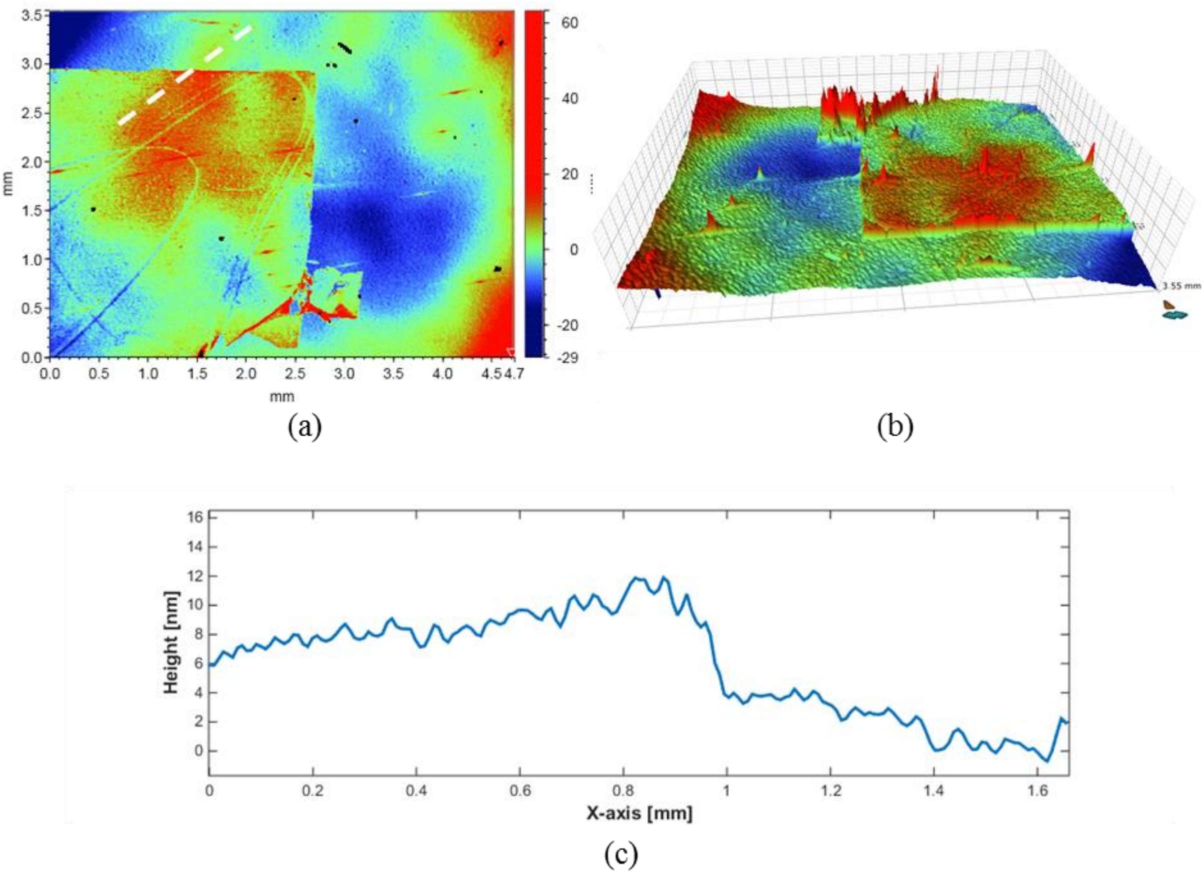

Figure S2 Micro-interferometry showing the 2D (a) and 3D (b) topography of the graphene sample after removal of the PMMA layer. The colours represent the scale of thickness from approximately 60 nm (red) to -29 nm (blue) (the point at which the zero level was specified is arbitrary and the peak maximum and minimum are dominated by the edge of the film and the surface structure of the substrate). A representative cross section, shown in white in (a) is plotted in (c). The thickness of the graphene layer is approximately 6 +/- 2 nm, confirming removal of the PMMA.

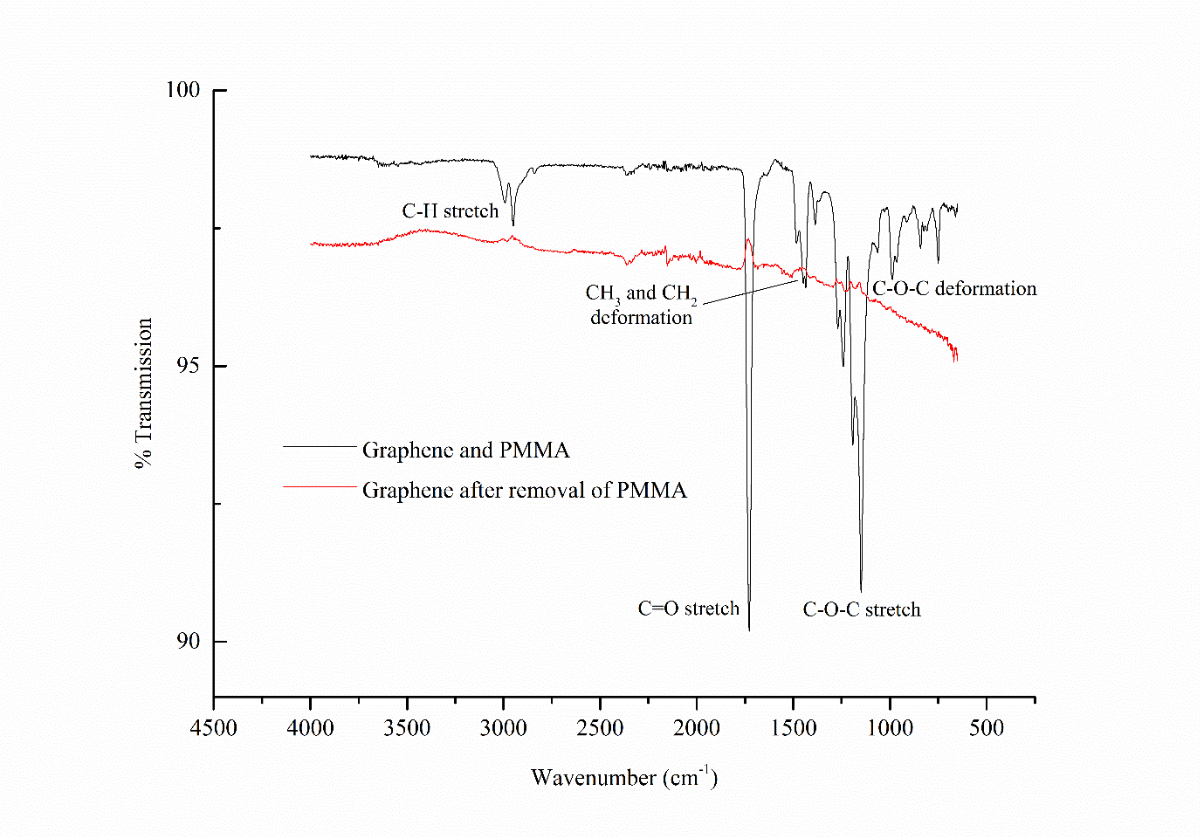

Figure S3 Infrared spectra from Trivial Transfer graphene before (black), showing the characteristic lines of an acrylic molecule, and after treatment with acetone (red) confirming removal of the PMMA from the graphene.

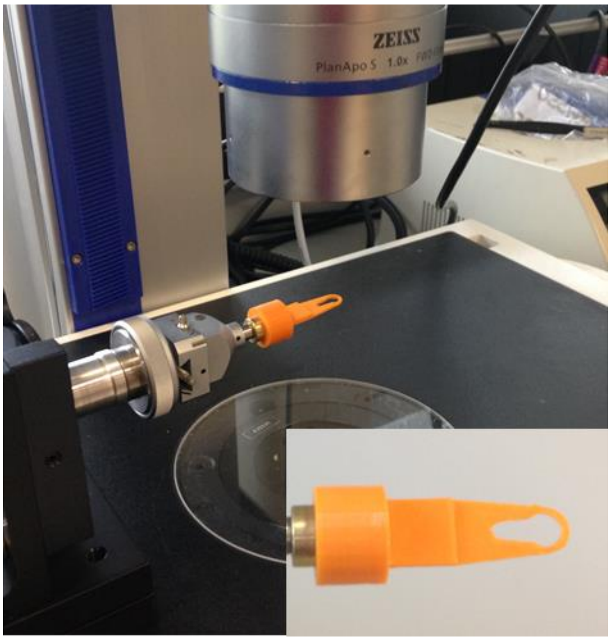

Figure S4 Image of the 3D-printed loop for mounting graphene/PMMA-wrapped crystals under the optical microscope. The image shows the set up under the optical microscope with the addition of a rotation stage for easy rotation of the sample. The inset shows a close-up of the 3D-printed mount.
